# Safety, pharmacokinetics and efficacy findings in an open-label, single-arm study of weekly paclitaxel plus lapatinib as first-line therapy for Japanese women with HER2-positive metastatic breast cancer

**DOI:** 10.1007/s10147-015-0832-5

**Published:** 2015-05-13

**Authors:** Kenichi Inoue, Katsumasa Kuroi, Satoru Shimizu, Yoshiaki Rai, Kenjiro Aogi, Norikazu Masuda, Takahiro Nakayama, Hiroji Iwata, Yuichiro Nishimura, Alison Armour, Yasutsuna Sasaki

**Affiliations:** Division of Breast Oncology, Saitama Cancer Center, 780 Komuro, Ina-machi, Kita-adachi-gun, Saitama, 362-0806 Japan; Department of Breast Surgery, Tokyo Metropolitan Cancer and Infectious Diseases Center Komagome Hospital, Tokyo, Japan; Department of Breast Oncology and Endocrine Surgery, Kanagawa Cancer Center, Yokohama, Kanagawa Japan; Hakuaikai Medical Corporation, Sagara Hospital, Kagoshima, Japan; Department of Surgery, National Hospital Organization Shikoku Cancer Center, Matsuyama, Japan; Department of Surgery, Breast Oncology, National Hospital Organization Osaka National Hospital, Osaka, Japan; Department of Breast and Endocrine Surgery, Osaka University Graduate School of Medicine, Osaka, Japan; Department of Breast and Endocrine Surgery, Osaka Medical Center for Cancer and Cardiovascular Diseases, Osaka, Japan; Department of Breast Oncology, Aichi Cancer Center Hospital, Nagoya, Japan; Development and Medical Affairs Division, GlaxoSmithKline K.K., Tokyo, Japan; Research and Development, GlaxoSmithKline, Philadelphia, PA USA; Department of Medical Oncology, Saitama Medical University International Medical Center, Saitama, Japan; Division of Medical Oncology, Department of Medicine, Showa University Hospital, Tokyo, Japan

**Keywords:** Lapatinib, Paclitaxel, HER2, Metastatic breast cancer

## Abstract

**Background:**

Lapatinib is the human epidermal growth factor receptor 2 (HER2) targeting agent approved globally for HER2-positive metastatic breast cancer (MBC). The efficacy, safety and pharmacokinetics (PK) of lapatinib combined with paclitaxel (L+P) were investigated in this study, to establish clear evidence regarding the combination in Japanese patients.

**Methods:**

In this two-part, single-arm, open-label study, the tolerability of L+P as first-line treatment in Japanese patients with HER2-positive MBC was evaluated in six patients in the first part, and the safety, efficacy and PK were evaluated in a further six patients (making a total of twelve patients) in the second part. Eligible women were enrolled and received lapatinib 1500 mg once daily and paclitaxel 80 mg/m^2^ weekly for at least 6 cycles.

**Results:**

The only dose-limiting toxicity reported was Grade 3 diarrhea in one patient. The systemic exposure to maximum plasma concentration and area under the plasma concentration curve (AUC) for lapatinib, as well as the AUC of paclitaxel, were increased when combined. The most common adverse events (AEs) related to the study treatment were alopecia, diarrhea and decreased hemoglobin. The majority of drug-related AEs were Grade 1 or 2. The median overall survival was 35.6 months (95 % confidence interval 23.9, not reached). The response rate and clinical benefit rate were both 83 % (95 % confidence interval 51.6, 97.9).

**Conclusions:**

The L+P treatment was well tolerated in Japanese patients with HER2-positive MBC. Although the PK profiles of lapatinib and paclitaxel influenced each other, the magnitudes were not greatly different from those in non-Japanese patients.

## Introduction

Overexpression or gene amplification of human epidermal growth factor receptor 2 (HER2) occurs in approximately 20 % of breast cancer cases. It is known to be an independent prognostic factor that shortens survival of patients, and is associated with high rates of cell proliferation and lymph node metastases [1, 2]. At present, the standard regimens used worldwide are the trastuzumab-containing regimens, which have significantly improved the prognosis for HER2-positive breast cancer [3, 4]. One of the key combination regimens containing trastuzumab is with taxane and is recommended by clinical practice guidelines globally [3–5]. However, the majority of patients experience tumor recurrences and/or metastases and, hence, further treatment options for primary care as well as in the metastatic setting are required. Another important issue is the occurrence of brain metastases due to the inability of trastuzumab to cross the intact blood–brain barrier. Cardiotoxicity has been recognized as one of the important side-effects [6–11]. Lapatinib tosylate hydrate is a small molecule that reversibly inhibits the activity of epidermal growth factor receptor (EGFR) and HER2 tyrosine kinases. Lapatinib was first approved in the USA in 2006 in combination with capecitabine for HER2-positive metastatic breast cancer (MBC) which persisted or recurred after anthracycline, taxane and trastuzumab treatment. The approved condition is the same worldwide, including in Japan. Lapatinib has been evaluated in other combinations, such as trastuzumab, aromatase inhibitor and paclitaxel. Its combination with paclitaxel is approved in some countries/regions as first-line treatment for HER2-positive MBC patients in whom trastuzumab is not appropriate.

Lapatinib is a small molecule that passes through the compromised blood–brain barrier and is suggested to exert anti-tumor activity in metastatic brain lesions [12, 13]. This characteristic approach of lapatinib led to the development of lapatinib in combination with paclitaxel as first-line therapy of HER2-positive MBC, as a replacement for the trastuzumab regimens.

The first Phase I study of a lapatinib and paclitaxel combination (L+P) in HER2-positive MBC patients was conducted outside Japan. This study demonstrated the tolerability of lapatinib (1500 mg/day) in combination with both weekly paclitaxel 80 mg/m^2^ and with tri-weekly paclitaxel 135, 175, 200 and 225 mg/m^2^ [14]. Following this, the combination of lapatinib (1500 mg/day) and tri-weekly paclitaxel (175 mg/m^2^) as first-line therapy was evaluated in a Phase III study, in which 579 HER2-negative or unknown, advanced or recurrent breast cancer patients were enrolled [15]. Of 579 patients enrolled, 83 had HER2-positive MBC, and the combination regimen showed significant improvement in progression-free survival (PFS) compared to paclitaxel monotherapy [hazard ratio (HR) = 0.49; 95 % confidence interval (CI) 0.3–0.8; *p* = 0.008]. In another Phase III study that was mainly conducted in China and South America, the combination of lapatinib (1500 mg/day) with weekly paclitaxel (80 mg/m^2^) as first-line therapy was evaluated in 444 patients with HER2-positive MBC [16]. The overall survival (OS) of the combination was prolonged compared to paclitaxel monotherapy (HR = 0.74; 95 % CI 0.58–0.94; *p* = 0.0124). The combinations of lapatinib (1250 mg/day) or trastuzumab either with paclitaxel or docetaxel as first-line therapy for HER2-positive MBC were evaluated in a global Phase III study including Japan, which was conducted mainly in the USA/EU [17]. The study enrolled 636 patients; the results, reported in 2012, showed that the PFS of the lapatinib combination was not significantly different compared with the trastuzumab regimen [HR = 1.33 (95 % CI 1.06–1.67); *p* = 0.01].

In order to extrapolate the data from the above studies conducted outside Japan to clinical practice in Japan, we have conducted this study to investigate the tolerability, safety and pharmacokinetics (PK) of a lapatinib 1500 mg and weekly paclitaxel combination.

## Patients and methods

### Patients

Eligible patients were Japanese women older than 18 years with histologically confirmed invasive HER2-positive MBC, which was defined as immunohistochemistry (IHC) 3+ or fluorescence in situ hybridization (FISH)-positive (an amplification ratio ≥2.2) evaluated by a local laboratory. Patients were required to have at least one measurable lesion according to the Response Evaluation Criteria in Solid Tumors (RECIST) version 1.0 [18]. Patients must have received no prior therapy for metastatic diseases. Additional inclusion criteria were: Eastern Cooperative Oncology Group performance status of 0 or 1; left ventricular ejection fraction (LVEF) within the institutional normal range (or ≥50 %, if unavailable); adequate renal, hepatic and hematologic functions.

Major exclusion criteria were: prior therapy with an EGFR and/or HER2 inhibitor other than trastuzumab; unresolved/unstable, serious toxicity from prior therapy with investigational drug and/or anticancer treatment; uncontrolled infection; ≥Grade 2 peripheral neuropathy according to National Cancer Institute Common Terminology Criteria for Adverse Events (NCI-CTCAE) version 3.0; malabsorption syndrome or other conditions that would prevent the efficacy and safety evaluation of the study regimen. All patients provided written informed consent. The study was conducted in accordance with good clinical practice and all applicable regulatory requirements, including the Declaration of Helsinki.

### Study design

This was a single-arm, two-part, open-label Phase I/II study (ClinicalTrials.gov Identifier: NCT01138046). The objectives were to evaluate the tolerability, safety, efficacy and PK of L+P in Japanese patients with HER2-positive MBC who have not received prior chemotherapy or trastuzumab for metastatic diseases. Six patients were enrolled in the first part (Part 1) to evaluate the tolerability of this regimen, and once the tolerability was confirmed, a further 6 patients were enrolled in the second part (Part 2). Sample size was not decided by statistical considerations.

Patients were given the standard treatment consisting of 6 cycles of weekly paclitaxel 80 mg/m^2^ (for 3 weeks in a 4-week cycle) combined with lapatinib 1500 mg daily; the latter was given until disease progression or withdrawal from study treatment due to unacceptable toxicity or consent withdrawal. The investigators could chose to continue concurrent paclitaxel administration for more cycles. For patients enrolled in Part 1, lapatinib was not given on day 1, but started on the following day, and paclitaxel was not given on day 15, but was given within 2 days after that date during the first cycle for PK evaluation purposes. If disease progression, unacceptable paclitaxel-related toxicities, or termination of lapatinib occurred, then paclitaxel was terminated at any time during the study, even before completing 6 cycles.

### Study endpoints

The objective in Part 1 was tolerability. If one or no patient out of the 6 experienced any events included in the tolerability criteria, the study treatment was concluded to be tolerable. PK parameters were also evaluated.

The efficacy and safety of the treatment in twelve patients were the objectives of Part 2. The study was not designed based on statistical hypotheses, as the study targeted a small population and was designed as single-arm stud;, however, in order to compare with the OS of the previous study, our efficacy evaluation was primarily focused on OS. The other endpoints of efficacy were PFS, time to response, duration of response, objective tumor response rate (ORR) and clinical benefit rate [complete response (CR) or partial response (PR) or stable disease (SD) ≥24 weeks]. Safety and biomarkers were also evaluated. All these endpoints were evaluated in the patients enrolled in both parts.

### Safety and efficacy assessments

Safety assessment including laboratory tests were performed every week during the combination treatment and at the discontinuation of paclitaxel if it was decided to continue longer than 6 cycles. If paclitaxel was discontinued at the sixth cycle, then safety assessment was conducted every 8 weeks until the end of treatment. LVEF assessment by echocardiogram was performed at the end of even-numbered cycles during combination treatment and every 8 weeks while on lapatinib monotherapy. Adverse events (AEs) were graded according to NCI-CTCAE version 3.0. AE terms were coded by MedDRA Ver13.1. The protocol defined serious AEs as all Grade 4 laboratory abnormalities, Grade 3 or 4 decrease in LVEF, ≥20 % decrease in LVEF relative to baseline and also below the institution’s lower limit of normal (if the lower limit of normal was unavailable, decrease to less than 50 %), Grade 3 pneumonitis, alanine aminotransferase (ALT) >3 × upper limit of normal (ULN), and total bilirubin >2.0 × ULN (>35 % direct; bilirubin fractionation required). The tolerability criteria were defined as the toxicities related to study treatment and applicable to any of the following: Grade 4 neutropenia sustained for ≥7 days, Grade 4 thrombocytopenia, ≥Grade 3 or clinically significant non-hematologic toxicities (other than nausea), or unable to start cycle 2 within 2 weeks of scheduled dosing due to unresolved toxicity. Patients withdrawn from the study without disease progression were assessed every 12 weeks until progression, start of post-anticancer therapy or death. Efficacy assessment was performed at baseline and at the ends of every even-numbered cycle until withdrawal from the study. Tumor response was assessed by the investigators, using images or photographic data, in accordance with the RECIST [18]. Biomarker analysis for HER2 status was conducted based on the results determined by both IHC and FISH at the central laboratory.

### Pharmacokinetics evaluation

Pharmacokinetics of lapatinib and/or paclitaxel were evaluated in all patients enrolled in Part 1 on days 1, 8 and 14: day 14 for PK of lapatinib monotherapy, day 1 for paclitaxel monotherapy and day 8 for combination therapy. Plasma samples were taken pre-dose and at 0.5, 1, 1.5, 2, 3, 4, 6, 8, 12 and 24 h post-dose. The PK parameters calculated by non-compartmental analysis were maximum plasma concentration (*C*_max_), time to *C*_max_ (*t*_max_) and area under the plasma concentration curve (AUC) from 0 to 24 h (AUC_0–24_) of lapatinib as well as *C*_max_, *t*_max_, half-life, AUC extrapolated to infinity (AUC_0–inf_) and AUC_0–24_ of paclitaxel.

### Statistical analysis

The sample size was determined based on study feasibility. This study did not assert or test any statistical hypotheses.

The dose-limiting toxicity (DLT) incidences were evaluated in 6 patients. The intent-to-treat (ITT) population was analyzed for safety and efficacy analyses. PFS and OS were summarized using the Kaplan–Meier method. All the patients who had provided ample plasma samples for the PK parameter evaluation were treated as the PK population. For PK parameters, the items evaluated are shown in Table 5.

Version 9.1.3 Unix SAS^®^ system (a registered trademark of the SAS Institute, Inc., Cary, NC, USA) was used for analysis.

## Results

### Patient characteristics

A total of 12 patients were enrolled from 9 centers between April 2010 and June 2011, and were treated with the study regimen. As of 31 January 2014 (the final data cut-off date), 6 patients had completed the study and 6 patients were followed up for survival.

Out of 12 patients enrolled, 8 patients had both visceral and non-visceral metastatic lesions, 2 patients had visceral lesions only, while the other 2 patients had non-visceral lesions only (Table 1). The median time since diagnosis was 12.9 months; 4 patients had received prior chemotherapy, of whom one had received prior trastuzumab. Six patients had estrogen receptor (ER)-positive breast cancer as assessed by a local laboratory, of whom 4 patients were positive for both ER and progesterone receptor.

**Table 1:** Baseline characteristics of intent-to-treat population

Age, years	
Median (range)	59.0 (45–70)
Time since diagnosis (months)	
Median (min–max)	12.9 (0–115)
1st Quartile	1.2
3rd Quartile	76.4
Prior anti-cancer therapy, *n* (%)	
Chemotherapy	4 (33)
Anthracyclines	1 (8)
Taxanes	3 (25)
Trastuzumab	1 (8)
Surgery	6 (50)
Radiotherapy	2 (17)
Endocrine therapy	4 (33)
Immunotherapy	0
Metastatic sites, *n* (%)	
Visceral	2 (17)
Non-visceral	2 (17)
Visceral and non-visceral	8 (67)
Hormone receptor status, *n* (%)	
ER+ and/or PgR+	6 (50)
ER+ and PgR+	4 (33)
ER+ and PgR−	2 (17)
ER− and PgR−	6 (50)
Unknown	0

Based on diagnosis made by investigators

*ER* estrogen receptor, *PgR* progesterone receptor

### Tolerability and safety

The median duration of lapatinib treatment was 50.9 weeks (range 4–117 weeks). Toxicities other than hematologic or neurologic toxicities leading to dose reduction occurred in 4 patients; however, DLT was not observed. The numbers of dose reductions observed were once (1250 mg) in 2 patients, twice (1000 mg) in 1 patient and three times (750 mg) in 1 patient. The primary reasons for dose reduction were rash, acne, diarrhea, increased ALT and increased aspartate aminotransferase (AST). Dose interruptions of lapatinib were reported in 10 patients, 73 times in total, mainly due to hematologic or neurologic toxicities. The median duration of interruption was 7 days (range 1–21 days).

For paclitaxel, the median number of cycles was 10 cycles (range 2–36 cycles), in which eight patients received more than 6 cycles. Neurologic toxicity was the cause of the dose reduction in one patient and of the dose interruptions of paclitaxel in 10 patients. All 12 patients were withdrawn from L+P, mostly due to disease progression.

In Part 1, the tolerability and safety of the study treatment in Japanese patients were confirmed. All patients experienced at least one AE regardless of the relationship with the study treatments, and most of them were at Grades 1 or 2. The most common AEs reported were alopecia, neutropenia, diarrhea, decreased hemoglobin and rash (Table 2). Grade 3 treatment-related AEs found in more than 2 patients were: neutropenia (*n* = 7), leukopenia (*n* = 5), diarrhea (*n* = 3), increased ALT (*n* = 3) and increased AST (*n* = 2). A Grade 4 treatment-related event, neutropenia, occurred in 2 patients.

**Table 2:** Summary of adverse events with at least 50 % occurrence

Adverse event, *n* (%)	Grade 1	Grade 2	Grade 3	Grade 4	Total
Alopecia	6 (50)	6 (50)	0	0	12 (100)
Diarrhea	4 (33)	4 (33)	3 (25)	0	11 (92)
Neutropenia	0	2 (17)	7 (58)	2 (17)	11 (92)
Decreased hemoglobin	3 (25)	6 (50)	1 (8)	0	10 (83)
Rash	6 (50)	3 (25)	0	0	9 (75)
Stomatitis	8 (67)	0	0	0	8 (67)
Fatigue	8 (67)	0	0	0	8 (67)
Peripheral sensory neuropathy	6 (50)	2 (17)	0	0	8 (67)
Leukopenia	0	3 (25)	5 (42)	0	8 (67)
Decreased apatite	4 (33)	3 (25)	0	0	7 (58)
Paronychia	5 (42)	2 (17)	0	0	7 (58)
ALT Increased	0	4 (33)	3 (25)	0	7 (58)
AST Increased	1 (8)	3 (25)	2 (17)	0	6 (50)
Lymphopenia	1 (8)	4 (33)	1 (8)	0	6 (50)
Decreased hematocrit	5 (42)	1 (8)	0	0	6 (50)
Vomiting	4 (33)	2 (17)	0	0	6 (50)
Nasopharyngitis	5 (42)	1 (8)	0	0	6 (50)
Nail disorder	4 (33)	2 (17)	0	0	6 (50)

*ALT* alanine aminotransferase, *AST* aspartate aminotransferase

Rash and diarrhea were the special interest AEs for lapatinib. No ≥Grade 3 or serious rash was reported. One Grade 2 rash event led to withdrawal from study treatment in one patient who had concurrently experienced Grade 2 erythema of the eyelid and on the back of both hands. Although Grade 3 diarrhea events occurred in 3 patients, no diarrhea was reported as ≥Grade 4 or serious, and there was no withdrawal from study treatment due to diarrhea.

No fatal serious AE was reported. Four protocol-defined serious AEs were reported in 3 patients; these were decreased neutrophil count in 2 patients, left ventricular dysfunction in a patient with a history of prior anthracycline treatment for other past malignancy, and pneumonia in a patient who was diagnosed by X-ray imaging. All these were considered by investigators to be treatment-related. Although the follow-up of left ventricular dysfunction was discontinued due to the start of another treatment, other serious AEs resolved within 2 weeks.

### Efficacy

**Fig. 1:**
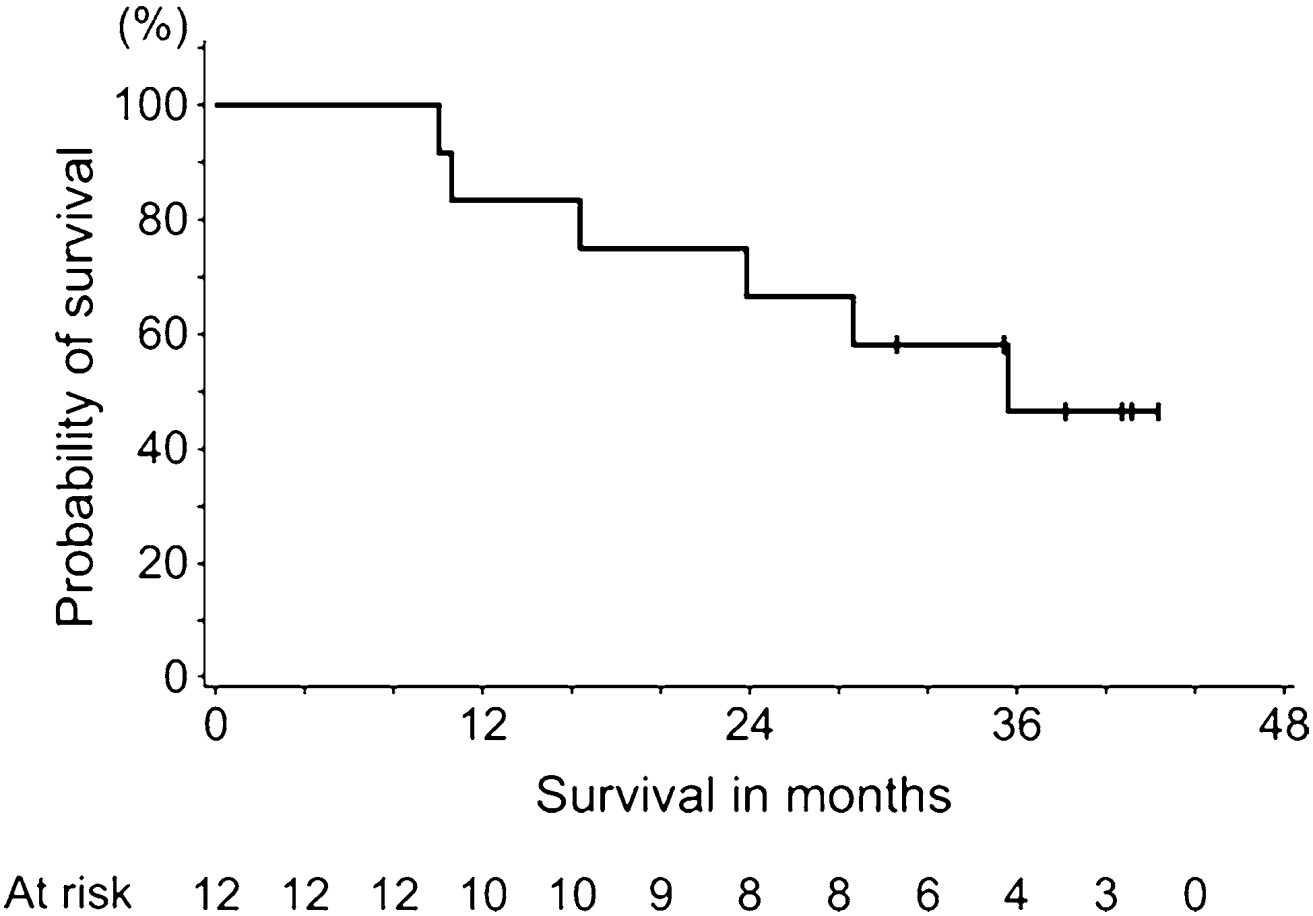
Kaplan–Meier estimates for overall survival

**Fig. 2:**
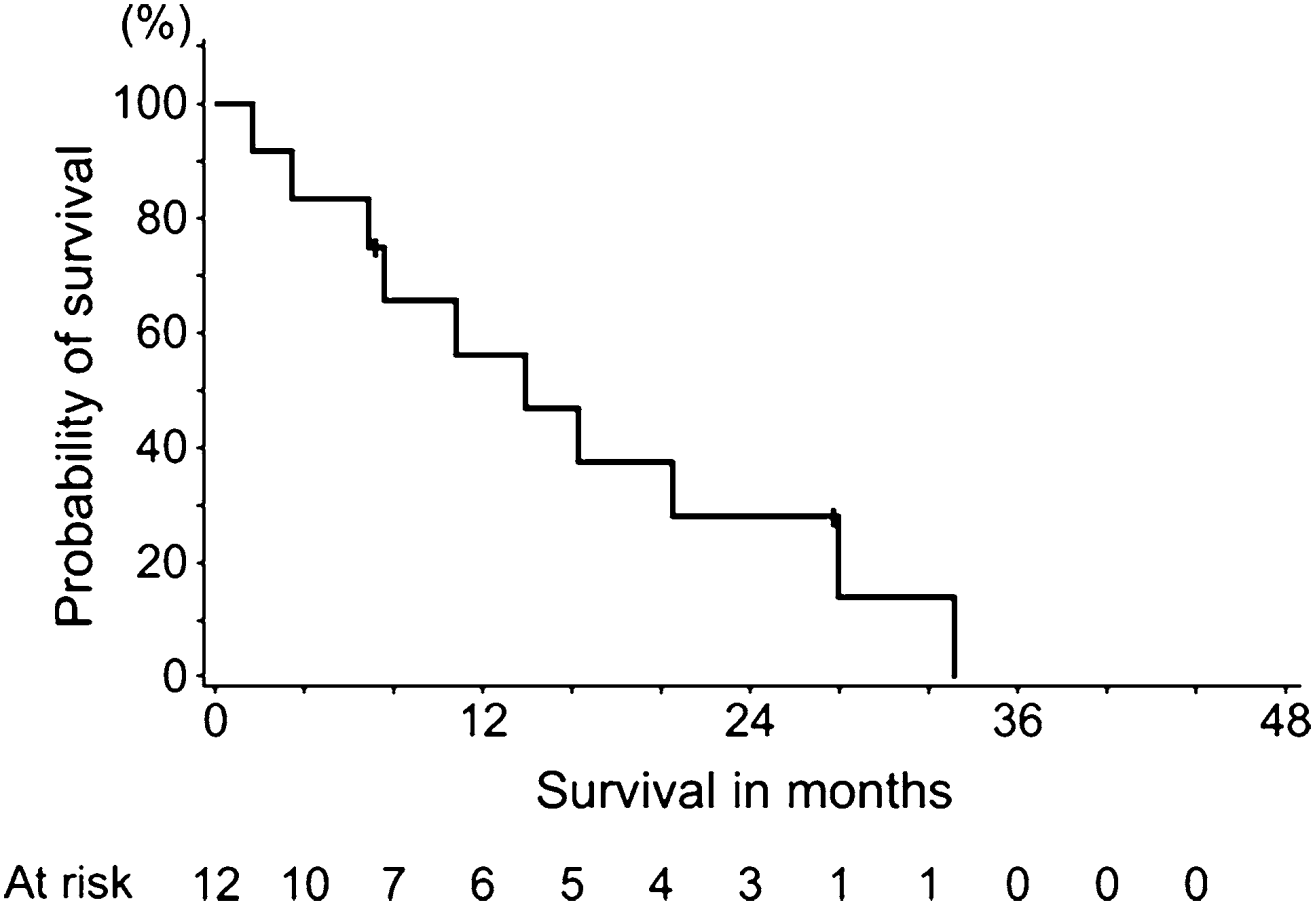
Kaplan–Meier estimates for progression-free survival assessed by investigators

As of the end of the study, 6 patients had died. The remaining 6 patients were censored at the last visit. The median OS as primary endpoint was 35.6 months (95 % CI 23.9, not reached; Fig. 1). PFS was analyzed using the results evaluated by the investigators and the median was 13.9 months (95 % CI 7.6, 27.9; Fig. 2).

Ten out of 12 patients (83 %) achieved clinical benefit (95 % CI 51.6, 97.9) based on the investigators’ assessment (Table 3). The ORR in the ITT population was 83 % (95 % CI 51.6, 97.9) with 10 PRs, while one patient had SD for less than 24 weeks and progressive disease was observed in one patient.

**Table 3:** Summary of tumor response in intent-to-treat population

Best response, *n* (%)	
CR	0
PR	10 (83)
SD, ≥24 weeks	0
SD, <24 weeks	1 (8)
PD	1 (8)
NE	0
ORR	83 (95 % CI 51.6, 97.9)
CBR	83 (95 % CI 51.6, 97.9)

*CBR* clinical benefit rate (CR; PR; SD ≥24 weeks), *CR* complete response, *NE* not evaluable, *ORR* overall tumor response rate, *PD* progressive disease, *PR* partial response, *SD* stable disease

### Pharmacokinetics

**Fig. 3:**
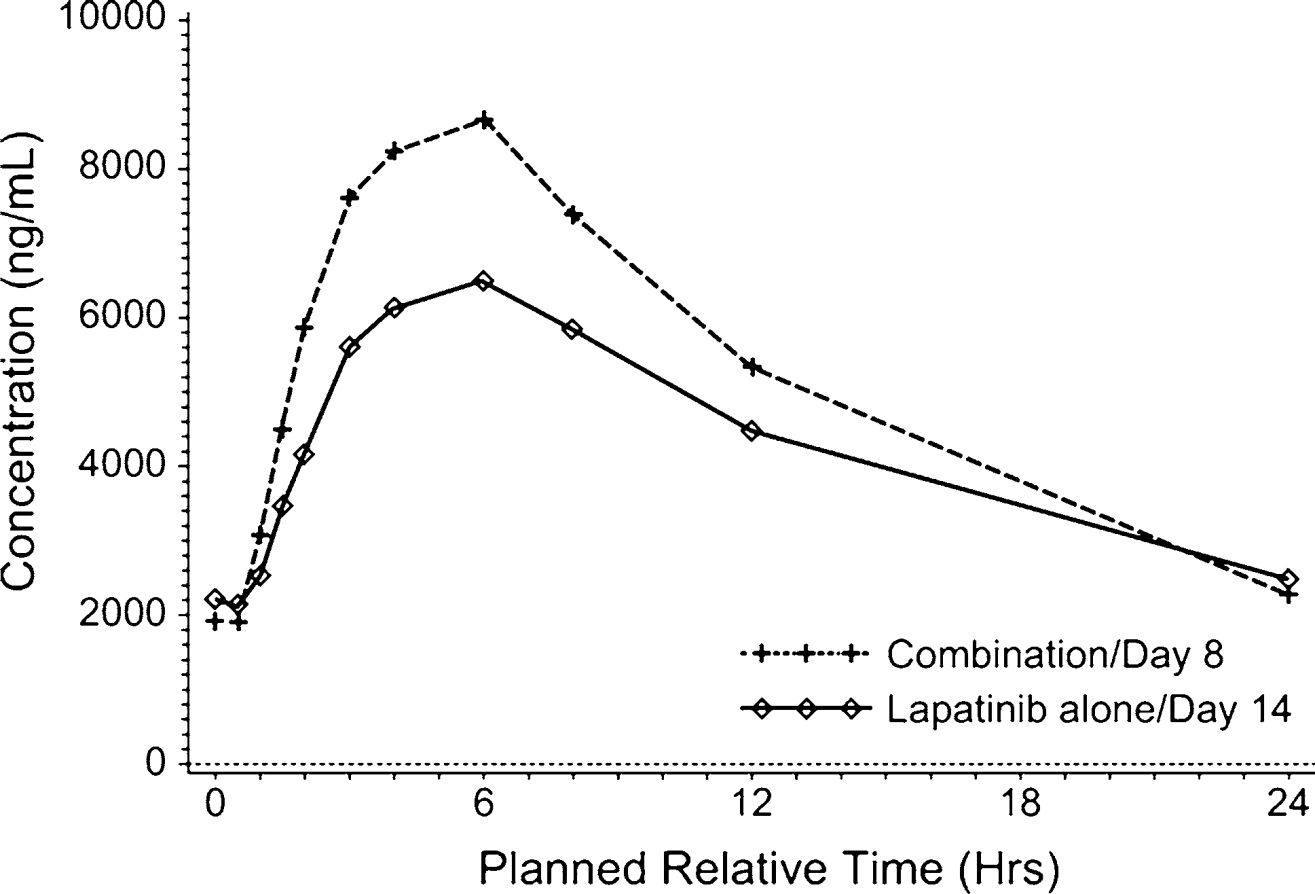
Plasma concentration–time profile of lapatinib after dosing of lapatinib 1500 mg with or without concomitant administration of paclitaxel 80 mg/m^2^

**Fig. 4:**
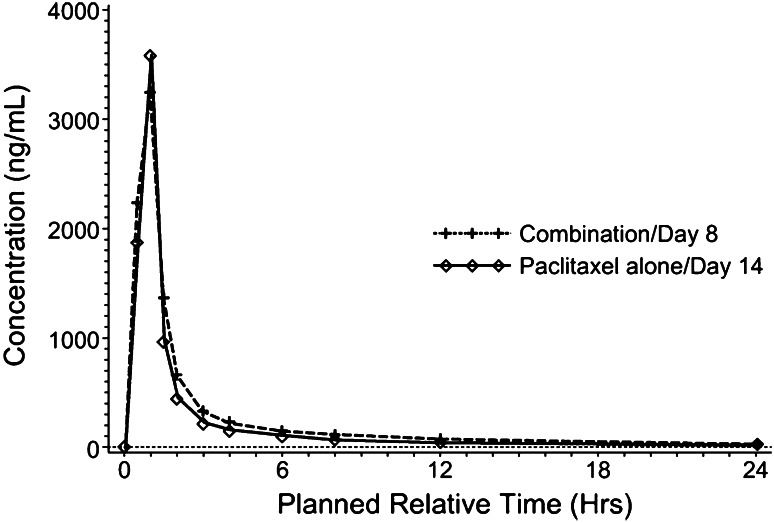
Plasma concentration–time profile of paclitaxel after dosing of paclitaxel 80 mg/m^2^ with or without concomitant administration of lapatinib 1500 mg

The plasma concentration–time profile of lapatinib after repeated oral dosing of lapatinib 1500 mg with or without concomitant administration of paclitaxel is shown in Fig. 3, and the plasma concentration–time profile of paclitaxel after 1 h intravenous infusion of paclitaxel 80 mg/m^2^ with or without concomitant administration of lapatinib is shown in Fig. 4.

The geometric means of *C*_max_ and AUC_(0–24)_ of lapatinib were increased by 59 and 42 % under the paclitaxel 80 mg/m^2^ combination, in comparison to lapatinib alone. No change was observed in *t*_max_ (Table 4). The geometric means of *C*_max_ and *t*_max_ of paclitaxel were not changed by combination with lapatinib; however, that of AUC_(0–inf)_ was increased by 23 % (Table 5).

**Table 4:** Pharmacokinetic parameters of lapatinib after repeat dosing with or without concomitant administration of paclitaxel

Parameter	Lapatinib 1500 mg alone (*n* = 6)	Lapatinib 1500 mg + paclitaxel 80 mg/m^2^ (*n* = 6)	Ratio (90 % CI)^a^
*C*_max_ (ng/mL)	5945.0 (3077.9, 11483.0)	9470.4 (7157.9, 12530.0)	1.59 (0.96, 2.64)
*t*_max_ (h)	4.992^b^ (1.50, 6.08)^c^	4.975^b^ (1.95, 6.00)^c^	0.00^d^ (−2.06, 2.21)
AUC_(0–24)_ (ng.h/mL)	79518.0 (34664.6, 182408.5)	113078.3 (74630.1, 171334.3)	1.42 (0.82, 2.46)

Geometric mean (95 % CI)

*AUC*_*(0*–*24)*_ area under the curve from 0 to 24 h, *CI* confidence interval, *C*_*max*_ maximum plasma concentration, *t*_*max*_ time to reach maximum concentration

^a^Ratio = (lapatinib + paclitaxel)/lapatinib alone

^b^Median

^c^Min and max

^d^Median difference

**Table 5:** Pharmacokinetic parameters of paclitaxel after dosing with or without concomitant administration of lapatinib

Parameter	Paclitaxel 80 mg/m^2^ alone (*n* = 6)	Paclitaxel 80 mg/m^2^ + lapatinib 1500 mg (*n* = 6)	Ratio^a^ (90 % CI)
*C*_max_ (ng/mL)	3485.2 (2693.0, 4510.5)	3412.3 (2753.1, 4229.4)	0.98 (0.85, 1.13)
*t*_max_ (h)	0.992^b^ (0.95, 1.08)^c^	0.975^b^ (0.50, 1.02)^c^	−0.05^d^ (−0.27, 0.00)
AUC_(0–24)_ (ng.h/mL)	4657.2 (3942.6, 5501.3)	5786.1 (4667.5, 7172.8)	1.24 (1.13, 1.36)
AUC_(0–inf)_ (ng.h/mL)	5125.9 (4371.3, 6010.8)	6280.0 (5005.6, 7878.8)	1.23 (1.10, 1.37)
*t*_1/2_ (h)	12.19 (10.06, 14.77)		9.86 (8.48, 11.46) 0.81 (0.71, 0.92)

Geometric mean (95 % CI)

*AUC*_*(0*–*24)*_ area under the curve, *AUC*_*(0*–*inf)*_ area under the curve extrapolated to infinity, *C*_*max*_ maximum plasma concentration, *CI* confidence interval, *t*_*max*_ time to reach maximum plasma concentration, *t*_*1/2*_ half-life

^a^Ratio = (paclitaxel + lapatinib)/paclitaxel alone

^b^Median

^c^Min and max

^d^Median difference

## Discussion

The tolerability of the lapatinib (1500 mg/day) and weekly paclitaxel (80 mg/m^2^) combination was confirmed in Japanese patients with HER2-positive MBC. Reported results from the pilot part of the Asian Phase III study targeting gastric cancer patients showed a similar tolerability of this regimen [19].

Drug–drug interaction of lapatinib and paclitaxel was found, as the AUC and *C*_max_ of Japanese breast cancer patients given the combination were affected. A similar trend of interaction was observed in a Phase I study targeting solid tumor patients conducted outside of Japan [14]. The extent of drug interaction of L+P was also similar to those of the pilot part of the Asian Phase III study which included patients with a history of gastrectomy [19]. The interaction found in PK profiles of lapatinib and paclitaxel is consistent regardless of the cancer type, and such interaction was considered to be the result of lapatinib being a weak metabolism-dependent inhibitor of CYP3A4.

The AEs reported in this study were generally consistent with those reported in overseas clinical studies evaluating L+P and with known safety profiles of lapatinib and paclitaxel given as monotherapy [14–17]. The grade of diarrhea was slightly worsened compared with lapatinib monotherapy, which means that L+P would require more careful management in clinical practice.

Our study demonstrated that the combination therapy of L+P was efficacious for the treatment of HER2-positive MBC, which was consistent with the results of a Phase III study [16]. Meanwhile, results became available from another global Phase III study which showed a significant PFS of a trastuzumab-containing regimen compared with a lapatinib and taxane regimen [17]. Moreover, the results of a trastuzumab, docetaxel and pertuzumab tri-regimen became available after the results of that global study were reported, and, up to 2014, this tri-regimen became the standard in first-line therapy of MBC worldwide [20]. As the new treatment of trastuzumab, pertuzumab and docetaxel tri-regimen became available for MBC and the results of direct comparison between lapatinib and trastuzumab were confirmed, it is now proven to be difficult to recommend L+P as the first-line therapy, which we originally expected.

Overall, our study provides valuable results that show the drug–drug interaction and PK interaction between lapatinib and paclitaxel in Japanese patients with MBC. Although our study does not impact upon the clinical positions or the treatment strategy, it confirms that the combination of lapatinib with paclitaxel is tolerable in Japanese patients with MBC. Nevertheless, paclitaxel remains a key drug in breast cancer therapies and many multiple-agent regimens with paclitaxel have been evaluated worldwide. For this, it is considered that our findings of PK and safety data of L+P may be beneficial to those who seek the appropriate dose and safety management of the regimen. Currently, studies targeting the adjuvant/neoadjuvant setting in which to evaluate combinations such as lapatinib, paclitaxel and trastuzumab are ongoing. There is no evidence to recommend the use of lapatinib in comparison to trastuzumab; however, our data can be utilized for considering the best practice of HER2 targeting therapies.

In conclusion, L+P was tolerable in Japanese patients with MBC, with manageable safety profiles, and a similar trend of the interaction of L+P to that previously reported in other ethnicities, as well as in different cancer types, was found.
